# Dangerous

**Published:** 1886-05

**Authors:** 


DANGEROUS, AND SHOULD BE PROHIBITED.

The talented chemist of the Texas State University, Prof. E. Everhart has been testing the coal oils sold and used in Austin, and of course, elsewhere in Texas, and pronounces them all as *dangerous* as *gun powder*; all of them flashing (and therefore exploding) at the *ordinary summer temperature of the atmosphere* in this climate. To-wit, Eupion oil flashes at 80° to 90°, Brilliant, 72° 80°, and none of them sold here will stand heat above 100°!

